# Shexiang Baoxin Pill, a Formulated Chinese Herbal Mixture, Induces Neuronal Differentiation of PC12 Cells: A Signaling Triggered by Activation of Protein Kinase A

**DOI:** 10.3389/fphar.2019.01130

**Published:** 2019-10-09

**Authors:** Miranda Li Xu, Zhong-Yu Zheng, Ying-Jie Xia, Etta Yun-Le Liu, Stanley Ka-Ho Chan, Wei-Hui Hu, Ran Duan, Tina Ting-Xia Dong, Chang-Sen Zhan, Xiao-Hui Shang, Karl Wah-Keung Tsim

**Affiliations:** ^1^Shenzhen Key Laboratory of Edible and Medicinal Bioresources, HKUST Shenzhen Research Institute, Shenzhen, China; ^2^Division of Life Science and Center for Chinese Medicine and State Key Laboratory of Molecular Neuroscience, The Hong Kong University of Science and Technology, Kowloon, Hong Kong; ^3^Shanghai Hutchison Pharmaceuticals Ltd, Shanghai, China; ^4^Shanghai Engineering Research Center for Innovation of Solid Preparation of TCM, Shanghai, China

**Keywords:** Alzheimer’s disease, Shexiang Baoxin Pill, Chinese medicine, neuronal differentiation, protein kinase A, cAMP-responsive element binding protein

## Abstract

**Background:** Shexiang Baoxin Pill (SBP) is a well-known composite formula of traditional Chinese medicine (TCM), which is commonly used today in treating cardiovascular diseases. SBP consists of seven materials thereof, including *Moschus*, extract of *Ginseng Radix et Rhizoma*, *Bovis Calculus Artifactus*, *Cinnamomi Cortex*, *Styrax*, *Bufonis Venenum*, and *Borneolum Syntheticum*. Here, we are investigating the potential roles of SBP in inducing neuron differentiation, i.e., seeking possible application in neurodegenerative diseases.

**Methods:** Water and ethanol extracts of SBP, denoted as SBP_water_ and SBP_EtOH_, respectively, as well as its individual herbal materials, were standardized and applied onto cultured rat pheochromocytoma PC12 cells. The potential effect of SBP extracts in neuronal differentiation was suggested by following parameters: (i) induction of neurite outgrowth of PC12 cells, (ii) increase of neurofilament expression, and (iii) activation of transcription of neurofilament.

**Results:** The treatments of SBP_water_ and SBP_EtOH_, or extracts from individual herbal materials, with or without low concentration of nerve growth factor (NGF), could potentiate the differentiation of cultured PC12 cells. The differentiation was indicated by increase of neurite outgrowth, as well as expression of neurofilaments. In addition, application of H89, a protein kinase A (PKA) inhibitor, suppressed the SBP-induced neurofilament expressions, as well as the phosphorylation of cAMP-responsive element binding protein (CREB) in cultures.

**Conclusion:** SBP is proposed to possess trophic activity in modulating neuronal differentiation of PC12 cells, and this induction is shown to be mediated partly by a cAMP-PKA signaling pathway. These results indicate the neurite-promoting SBP could be useful in developing potential drug in treating or preventing neurodegenerative diseases.

## Introduction

Progressive reduction of neuron is a pathologica#l hallmark of neurodegenerative diseases [e.g. Alzheimer’s disease (AD)]. Neurodegenerative disease is one of the major public health issues globally, with the particular increasing prevalence in old-age population (Blazer and Wallace, 2016). As of today, there is no effective treatment for neurodegenerative disease. Neurotrophic factors, also known as neurotrophins produced mainly in the brain, have been probed for therapeutic roles in neuronal diseases: these proteins are belonging to a group of growth factors in nurturing growth and survival of neurons, as such to maintain normal brain function (Weissmiller and Wu, 2012). Several lines of evidence suggest an insufficient supply of neurotrophic factors is leading to deduction in neuron, and thereafter causing neurodegenerative disorders (Cai et al., 2014). Therefore, neurotrophic factors have been identified as one of the potential therapeutic options for neurodegenerative disorders for a number of years (Weissmiller and Wu, 2012; Cai et al., 2014). However, short biological half-lives and inability to penetrate the blood-brain barrier (BBB) of protein factors greatly limit application of which in clinical usage. Therefore, the development of new compounds or drugs mimicking neurotrophic-like activity appears to be one of the promising therapeutic approaches in treating neurodegenerative diseases.

Traditional Chinese medicines (TCMs) have been widely used to prevent and cure various diseases in Asian countries for thousands of years. The record of TCM usage attributes to its effectiveness and relatively low toxicity (Yang et al., 2011). **S**hexiang **B**aoxin **P**ill (SBP), a well-known composite formula of TCM, is commonly used for treatment of cardiovascular diseases (Yan et al., 2006). Having clinical usage in the past 40 years, the therapeutic effects on stable angina pectoris and chest pain, caused by coronary heart disease, have been shown to be rather effective (Hong et al., 2006; Yan et al., 2006; He et al., 2009; Tian et al., 2011). The medicinal constituents of SBP includes: *Moschus*, extract of *Ginseng Radix et Rhizoma*, *Bovis Calculus Artifactus*, *Cinnamomi Cortex*, *Styrax*, *Bufonis Venenum* and *Borneolum Syntheticum*. The efficacy of SBP is highly recognized by its rapid action, and which possesses an accurate curative effects without obvious side effects (Lu et al., 2018). SBP has been officially recorded in 2010 edition of Chinese Pharmacopoeia (Xiang et al., 2012). Besides the function of SBP in cardiac disease, recent studies suggested potential roles of SBP in brain functions (Chen et al., 2008). Here, we speculated that SBP could possess neurogenic role in neuron. In cultured PC12 cells, the neurotrophic properties of water and ethanol extracts of SBP, as well as individual herbal materials in SBP, were analyzed for its inducing activity in neurite outgrowth. The effect of SBP extracts on neuronal differentiation was elucidated, including the signaling triggered by protein kinase A (PKA) and cAMP responsive element binding protein (CREB).

## Materials and Methods

### Chemical and Medicinal Materials

Four batches of SBP and seven medicinal materials or extracts, including *Moschus* (the dried secretion of musk sac of adult male *Moschus berezovskii*, *M. sifanicus*, or *M. moschiferus*; muscone is the main ingredient in *Moschus*), *ginseng radix et rhizoma* (supplied as the dried 75% ethanol extract of *Panax ginseng* root and rhizome, having over 0.27% ginsenoside Rg_1_ and ginsenoside Re and ginsenoside Rb_1_ not less than 0.18% by weight), *Bovis Calculus Artifactus* (prepared with powder of cow bile, cholic acid, hyodeoxycholic acid, taurine, bilirubin, cholesterol, trace elements, etc), *Cinnamomi Cortex* (the dried stem bark of *Cinnamomum cassia*), *styrax* (the acaroid resin obtained from the trunk of *Liquidambar orientalis* having over 5% cinnamic acid by weight). *Bufonis venenum* (the dried secretion of *Bufo bufo* gargarizans or *Bufo melanostitus*) and *Borneolum Syntheticum* (the synthetic crystal containing mainly borneol not less than 55% by weight). These herbal materials together with SBP extracts were obtained from Shanghai Hutchison Pharmaceuticals Company (Shanghai, China). The batch numbers of SBP were 170725, 171214, 180110, and 180112: the preparation of SBP was followed as described in Chinese Pharmacopeia 2015. The herbal materials were authenticated according to Chinese Pharmacopeia 2015, morphologically and chemically. The voucher species were stored at Center for Chinese Medicine at HKUST.

In preparing water (SBP_water_) and 95% ethanol (SBP_EtOH_) extracts, 20 g powders of SBP, *Moschus*, *Bovis Calculus Artifactus*, *Cinnamomi Cortex*, and *Bufonis Venenum* were sequentially sonicated twice in water or 95% ethanol in a proportion of 1:8 (w/#v; 160 ml) and 1:6 (w/v; 120 ml) for 30 min each time at 37°C. Total extracts were combined, dried under vacuum and stored at −80°C. The extracts of SBP_water_ were solubilized in H_2_O; while SBP_EtOH_ extracts, extract of *Ginseng Radix et Rhizoma* and *Borneolum Syntheticum* (synthetic having > 55% bornel) were dissolved in dimethylsulfoxide (DMSO). *Styrax* solution was prepared with DMSO in a ratio of 1:100 (v:v). These extractions were accord to preparative protocol of SBP. Stock solutions were at 100 mg/ml, stored at −20°C.

### HPLC Fingerprint

One g of SBP_water_ or SBP_EtOH_ was sonicated in 10 ml of EtOH. The extract was filtered; the supernatant was collected and analyzed for chemical fingerprint analysis. The analysis was performed on an Agilent HPLC 1200 system (Agilent, Waldbronn, Germany), equipped with a degasser, a binary pump, an auto sampler, a thermostatic column compartment, and a DAD. The samples were separated on a PLATISIL C_18_ column (4.6 mm × 250 mm, 5 μm i.d.) after filtered with a guard column. The mobile phase was composed of acetonitrile (A) and 0.03% phosphoric acid solution (B) according to pre-set gradient program: 0 to 25 min, linear gradient 15% to 40% (A), 85% to 60% (B); 25% to 55 min, linear gradient 40% to 75% (A), 60% to 25% (B); 55 to 65 min, linear gradient 75% to 100% (A), 25% to 0% (B); 65% to 75 min, 100% (A). The injection volume was 10 μl; the flow rate was 0.8 ml/min; and the column temperature was 25°C. The detector was set to 280 nm.

### Cell Culture and Herbal Treatment

Rat pheochromocytoma PC12 cell line (CRL-1721), derived from rat adrenal medulla, was obtained from American Type Culture Collection (Manassas, VA) and cultured in Dulbecco’s modified Eagle’s medium (DMEM), supplemented with 6% fetal bovine serum and horse serum, 100 units/ml penicillin, and 100 μg/ml streptomycin in a humidified CO_2_ (7.5%) incubator at 37°C. Fresh medium was applied every other day. Culture reagents were from Invitrogen (Carlsbad, CA). For drug treatment, PC12 cells after serum starvation for 3 h in DMEM containing 1% fetal bovine serum and horse serum were treated with NGF or herbal extract for 48 h. The cell viability was performed to determine a safe concentration range (0–500 μg/ml) of herbal extract, at which the extracts did not induce cell proliferation or death. The ethanol extracts of SBP and *Styrax* solution were used at 100 μg/ml; water extracts, Ginseng extract and *Borneolum Syntheticum* solution were used at 500 μg/ml. Cell viability was assessed by MTT assay. Cells were plated in 96-well plate for 24 h and treated with drugs for 48 h before adding MTT. Then, the cells were incubated with MTT for another 3 h at 37°C. After that, absorbance of 570 nm was measured in a microplate reader (Thermo Fisher Scientific, Waltham, MA) (Lam et al., 2016a; Lam et al., 2017).

### Neurite Outgrowth

Cultured PC12 cells were treated with herbal extracts with or without low concentration of NGF (1.5 ng/ml) for 48 h; fresh medium and reagents were supplied every 24 h. A light microscope (Zeiss Group, Jena, Germany), equipped with a phase-contrast condenser, 10× objective and a digital camera (Zeiss Group), was used to capture images with manual setting. To analyze number and length of neurite, approximately 100 cells were counted from at least 10 randomly chosen visual fields for each culture. By using SPOT basic software (Diagnostic Instruments, MI), cells were analyzed for length of neurite. The cells were scored as differentiated if one or more neurites was longer than diameter of cell body, and they were also classified to different groups according to length of neurite that it possessed, i.e. < 30 μm, 30 to 60 μm and > 60 μm (Yan et al., 2017).

### Western Blot and Phosphorylation

PC12 cells were seeded into 6-well plates in normal serum medium for 24 h and then transferred to low serum, as indicated for 3 h prior to exposure of SBP extracts in absence or presence of 5 µM H89 (Sigma, St. Louis, MO). After 48 h of treatment, the cultures were collected in high salt lysis buffer (1M NaCl, 10 mM HEPES, pH 7.5, 1 mM EDTA, 1 mM EGTA, 0.5% Triton X-100), followed by centrifugation at 16,100 X *g* for 10 min at 4°C. For SDS-PAGE, the protein lysate was denatured in presence of 2% SDS and 100 mM β-mercaptoethanol. Anti-NF200 (1:1,000, Sigma), anti-NF160 (1:2,500, Sigma), anti-NF68 (1:2,500, Sigma) and anti-GAPDH (1:10,000, Invitrogen) were used for Western blot analyses. For protein phosphorylation analysis, PC12 cells were seeded onto a 6-well plate. After the degree of confluence reached to > 90%, the culture medium was changed to DMEM without serum over 3 h. The cells were treated with NGF (50 ng/ml), SBP extracts, or ginsenoside Rg_1_ (10 μM) for different time (0, 5, 10, and 30 min). The phosphorylation of CREB was evaluated by immunoblotting with specific anti-phospho-CREB (1:2,500, Cell Signaling, Danvers, MA) and anti-total CREB antibodies (1:2,500, Cell Signaling). After intensive washing with Tris-buffer saline/0.1% Tween 20 (TBS-T), horseradish peroxidase (HRP)-conjugated anti-mouse, or anti-rabbit secondary antibody (Zymed, South San Francisco, CA), with a 1:5,000 dilution was added and incubated for 1 h at room temperature. After washing with TBS-T, the immuno-reactivity was detected by enhanced chemiluminescence (ECL) Western Blot System as stated by the manufacturer (GE Healthcare Life Sciences, Piscataway, NJ). The intensities of bands were quantified by ImageJ2x analysis software. The labeling intensities of protein bands were in the non-saturating range of calibration curves.

### DNA Construct and Transfection

Two DNA constructs of pNF68-Luc (promoter for NF68) and pNF200-Luc (promoter for NF200) reporter genes in pLightSwitch_Prom vector were purchased from Switchgear Genomics (Menlo Park, CA). Three repeats of cAMP response elements (CRE: 5’-TGA CGT CA-3’) were sub-cloned into pTAL vector (Clontech, Mountain View, CA) upstream of a firefly luciferase gene, designated as pCRE-Luc (Lam et al., 2016b). Transient transfection of PC12 cells with cDNA constructs was performed with a jetPRIME reagent (Polyplus Transfection, NY), according to the manufacturer’s instruction. The transfection efficiency was consistently 40% to 50% in PC12 cells, as determined by another control plasmid having a β-galactosidase gene under a cytomegalovirus (CMV) enhancer promoter.

### Other Assays

Luciferase assay was performed using a commercial kit (Thermo Fisher Scientific, Waltham, MA). In brief, cell cultures were washed with PBS and resuspended in 100 mM potassium phosphate buffer (pH 7.8) containing 0.2% Triton X-100 and 1 mM dithiothreitol (DTT). Forty μl of lysate per sample was used in luciferase assay. The luminescent reaction was quantified in a GloMax^®^ 96 Microplate Luminometer, and the activity was expressed as absorbance (up to 560 nm) per mg of protein. Protein concentrations were measured routinely by the Bradford method with a kit from Bio-Rad (Hercules, CA). Statistical tests were performed using one-way analysis of variance; differences from basal or control values were classified as * *p* < 0.05; ** *p* < 0.01; *** *p* < 0.001.

## Results

### Fingerprints of SBP

HPLC chromatograms of SBP_water_ (water extract of SBP) and SBP_EtOH_ (95% ethanol extract of SBP) were obtained, in which there were mainly 12 eluted peaks being identified in SBP_EtOH_; but only 3 of them were identified in SBP_water_ (**Supplementary Figure 1**). Thus, SBP_EtOH_ should contain more UV detectable chemicals than that of SBP_water_. The 12 peaks represented major constituents of SBP_EtOH_ extracts with consistent retention values (RSDs of retention times lower than 1%, and those of most peak areas lower than 8%). The HPLC chromatogram represented characteristic chemical information of non-volatile constituents in SBP. The authenticated 12 peaks, including peak 1 to 10, were confirmed by comparing retention times with the chemical standards. The HPLC chromatograms of water and 95% ethanol extracts of *Bufonis Venenum*, *Bovis Calculus*, *Moschus*, and *Cinnamomi Cortex* were obtained (**Supplementary Figure 1**). Furthermore, the HPLC chromatograms of extract of *Ginseng Radix et Rhizoma*, *Borneolum Syntheticum*, and *Styrax* were obtained (**Supplementary Figure 2**). Thus, the obtained HPLC chromatogram could be applied as fingerprint of SBP and other herbal extracts for quality control purpose.

### SBP Induces Neuronal Differentiation

**Figure 1:**
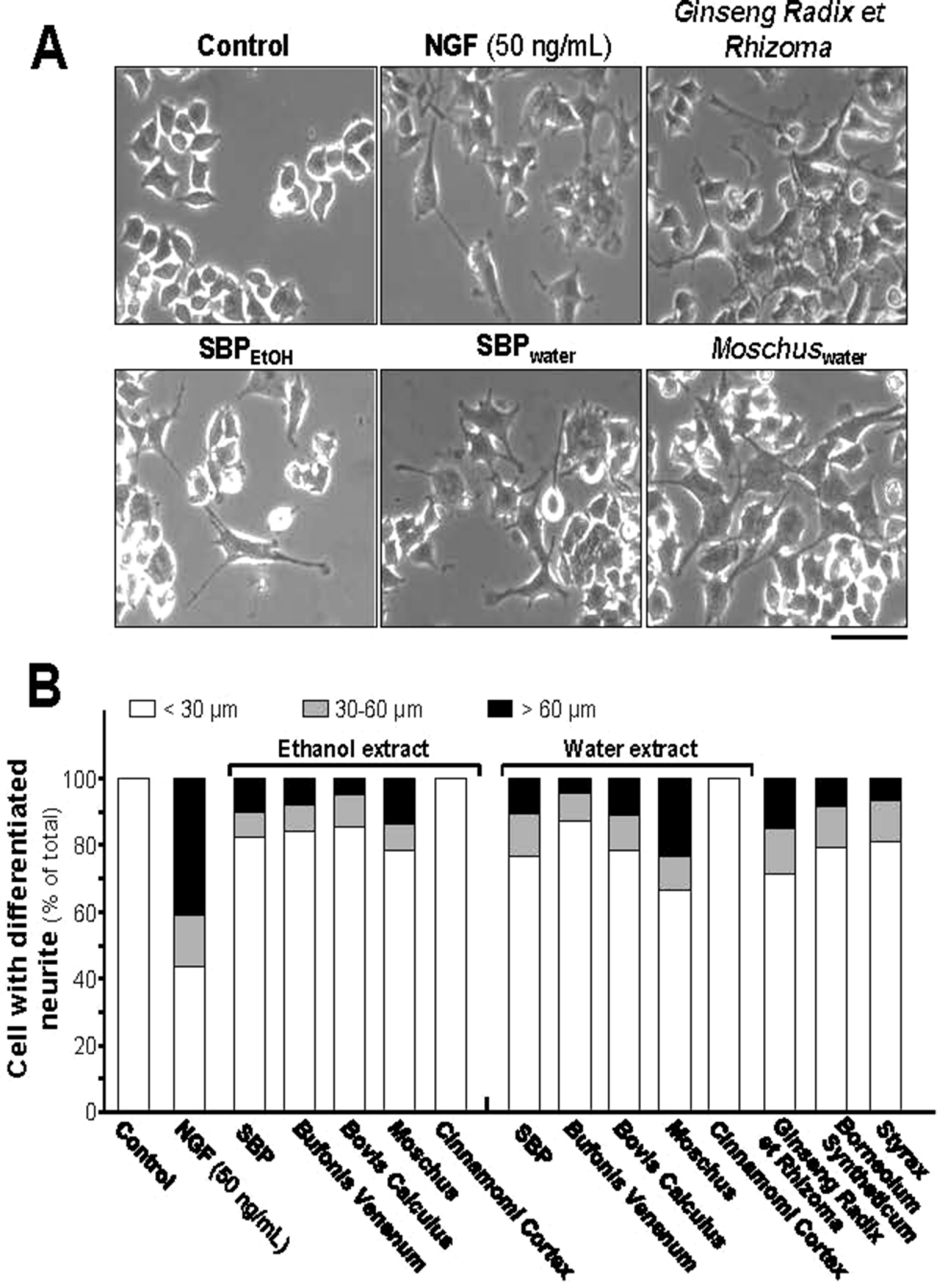
SBP extracts induce neurite outgrowth in PC12 cell. **(A)**: PC12 cells were treated with SBP_water_ and SBP_EtOH_, or individual herbal extracts for 48 h. NGF at 50 ng/ml served as a positive control. SBP_EtOH_, ethanol extracts of various herbal materials and *Styrax* solution were used at 100 μg/ml; and SBP_water_, water extracts of various herbal materials, solutions of *Ginseng Radix et Rhizoma* extract and *Borneolum Syntheticum* were used at 500 μg/ml. Scale bar = 100 μm. Representative images were shown. **(B)**: The lengths of neurite were counted as described in Method section. Values were expressed as percentage of cells having different length of neurite in 100 counted cells (total), mean ± SEM, *n* = 4.

Neurite elongation and branching of neuron are key cellular events during brain development as they underlie the formation of a properly wired neuronal network. PC12 cell is known to stop mitotic division and to differentiate under treatment of NGF: this is a firmly established model in analyzing the consequences during neuronal differentiation. The differentiation of PC12 cells could be determined morphologically in measuring the length of neurite, i.e., the neurite should be longer than diameter of cell body (Lee and Cleveland, 1996; Lam et al., 2016b). NGF at 50 ng/ml was employed as a control in cultured PC12 cells to stimulate neuronal differentiation, as well as the morphological change (**Figure 1A**). The direct inducible effect of herbal extracts in neurite outgrowth was determined in the absence of NGF. Neither water nor 95% ethanol extracts of *Cinnamomi Cortex* showed effect on neurite outgrowth. However, SBP_water_, SBP_EtOH_, the extracts of *Moschus*, *Ginseng Radix et Rhizoma*, *Bovis Calculus Artifactus*, *Styrax*, *Bufonis Venenum*, and *Borneolum Syntheticum* were able to induce neurite outgrowth (**Figures 1A, B**). The 95% ethanol extract of *Bufonis Venenum* showed stronger effect in inducing neurite outgrowth, as compared to its water extract. On the other hand, the water extracts of SBP, *Moschus*, and *Bovis Calculus Artifactus* showed stronger capability to induce neurite outgrowth rather than their ethanol extracts (**Figure 1B**). The extract of *Ginseng Radix et Rhizoma* and water extract of *Moschus* showed better effects in neurite outgrowth.

**Figure 2:**
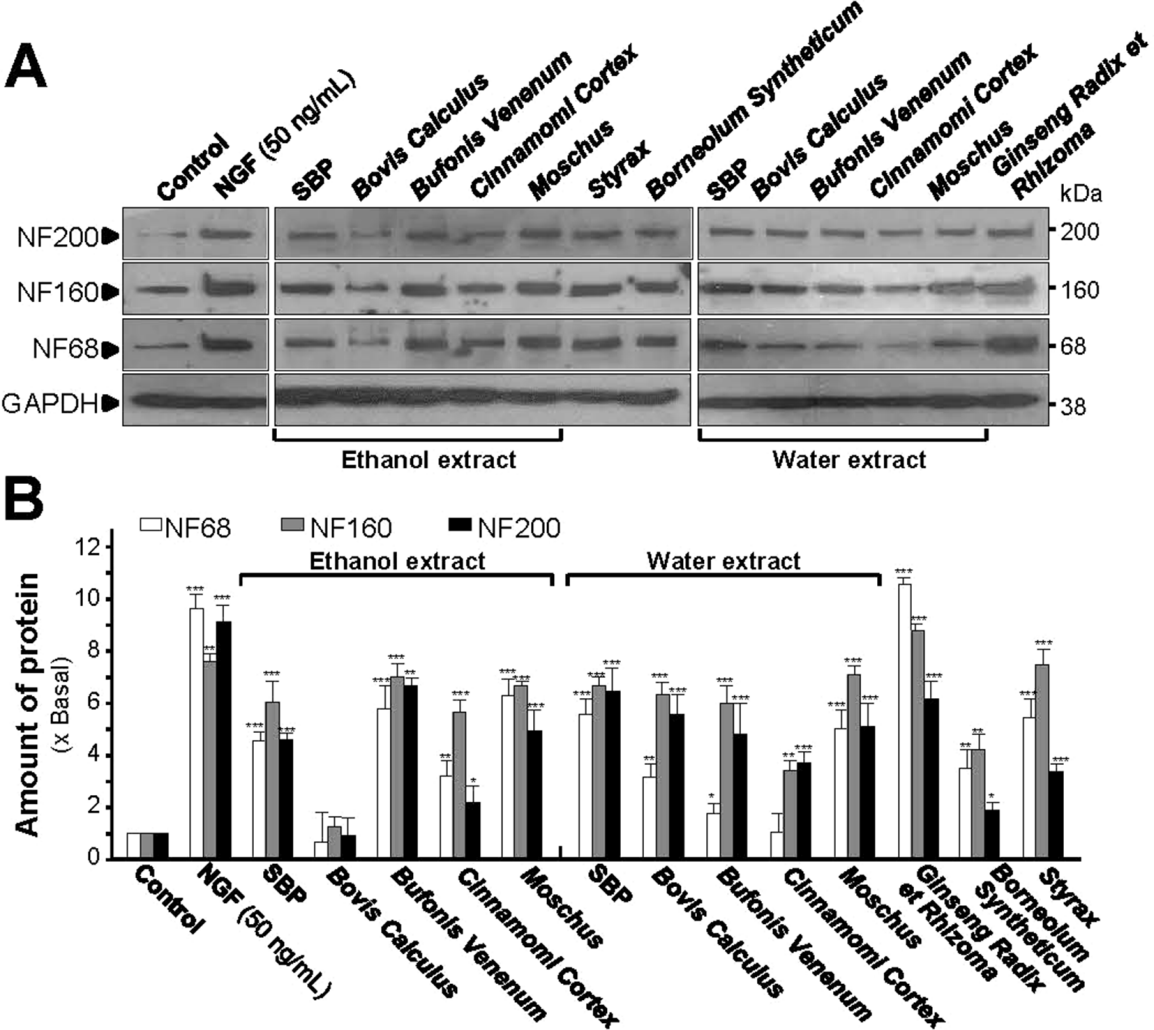
SBP extracts induce expression of neurofilaments. **(A)**: Cultured PC12 cells were treated with SBP_water_ and SBP_EtOH_, or individual herbal extracts for 48 h. NGF at 50 ng/ml served as a positive control. SBP_EtOH_, ethanol extracts of various herbal materials and *Styrax* solution were used at 100 μg/ml; and SBP_water_, water extracts of various herbal materials, solutions of *Ginseng Radix et Rhizoma* extract and *Borneolum Syntheticum* were used at 500 μg/ml. The cell lysates were collected to determine the expression of NF68 (∼68 kDa), NF160 (∼160 kDa), and NF200 (∼200 kDa). GAPDH (∼38 kDa) served as a loading control. **(B)**: Quantification from the blots by a densitometer was shown. Values were expressed as the fold of change (x Basal) against the control (no treatment; set as 1), mean ± SEM, *n* = 4, * *p* < 0.05; ** *p* < 0.01; *** *p* < 0.001.

The structural domain of neurite is composed of heterodimers of 3 mammalian neurofilament subunits, NF68 (∼68 kDa), NF160 (∼160 kDa) and NF200 (∼200 kDa) (Lee and Cleveland, 1996; Schimmelpfeng et al., 2004). For the expressions of neurofilaments, all extracts, except the ethanol extract of *Bovis Calculus Artifactus*, showed certain levels of protein induction. Both SBP_EtOH_ and SBP_water_ induced the expressions of NF68, NF160, and NF200 by 4 to 7 folds, significantly (**Figures 2A, B**). The strongest induction of neurofilaments was revealed by the extract of *Ginseng Radix et Rhizoma*, as good as that for NGF, a positive control (**Figures 2A, B**).

### SBP Promotes NGF-Induced Neuronal Differentiation

**Figure 3:**
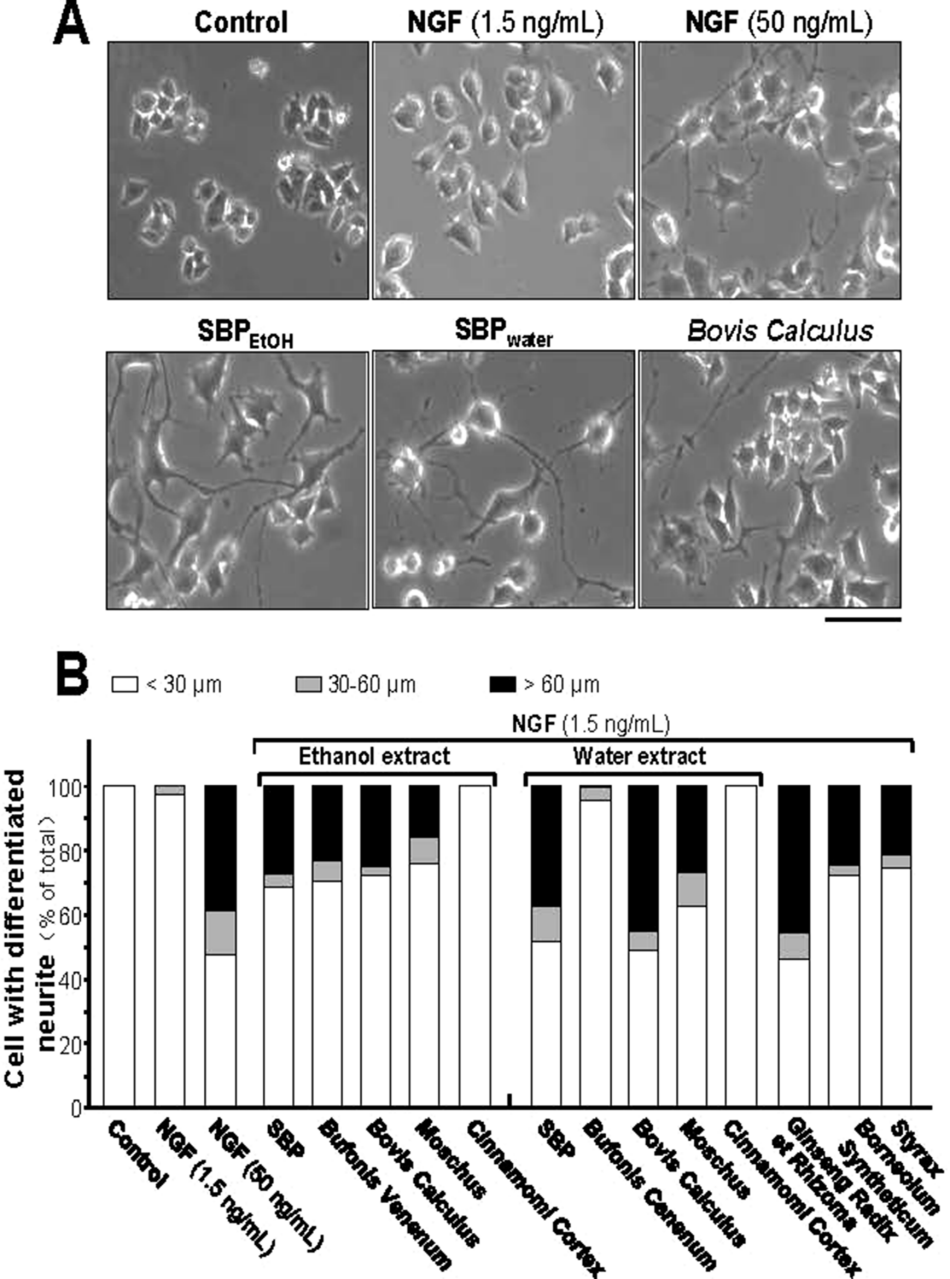
SBP extracts potentiate the NGF-induced neurite outgrowth in PC12. **(A)**: Cultured PC12 cells were co-treated with NGF (1.5 ng/ml) together with SBP_water_ and SBP_EtOH_, or individual herbal extracts for 48 h. NGF at 50 ng/ml served as a positive control. SBP_EtOH_, ethanol extracts of various herbal materials and *Styrax* solution were used at 100 μg/ml; and SBP_water_, water extracts of various herbal materials, solutions of *Ginseng Radix et Rhizoma* extract and *Borneolum Syntheticum* were used at 500 μg/ml. Scale bar = 100 μm. Representative images were shown. **(B)**: The lengths of neurite were counted as described in Method section. Values were expressed as percentage of cells having different length of neurite in 100 counted cells (total), mean ± SEM, *n* = 4.

Insufficiency of NGF in the brain is one of the causes of neurodegenerative diseases. Thus, we aimed to determine the efficacies of each treatment (i.e. SBP_water_ or SBP_EtOH_ and other seven individual herbal materials) together with low-dose of NGF (1.5 ng/ml): this NGF concentration failed to induce the neurite extension, and therefore which was chosen for the co-treatment analyses (**Figures 3A, B**). Neither water extract nor ethanol extract of *Cinnamomi Cortex* could synergize with low-dose of NGF in inducing neurite extension. In contrast, SBP_water_, SBP_EtOH_, extracts of *Moschus*, *Ginseng Radix et Rhizoma*, *Bovis Calculus Artifactus*, *Styrax*, *Bufonis Venenum*, and *Borneolum Syntheticum* were able to synergize with low-dose of NGF to induce neurite outgrowth at different degrees (**Figures 3A, B**). The ethanol extract of *Bufonis Venenum* showed much stronger effect in NGF-induced neurite outgrowth, as compared to its water extract. In general, the SBP_water_, *Bovis Calculus Artifactus*, and *Moschus* showed stronger capability to potentiate NGF-induced neurite outgrowth, instead of its corresponding ethanol extracts (**Figure 3B**).

**Figure 4:**
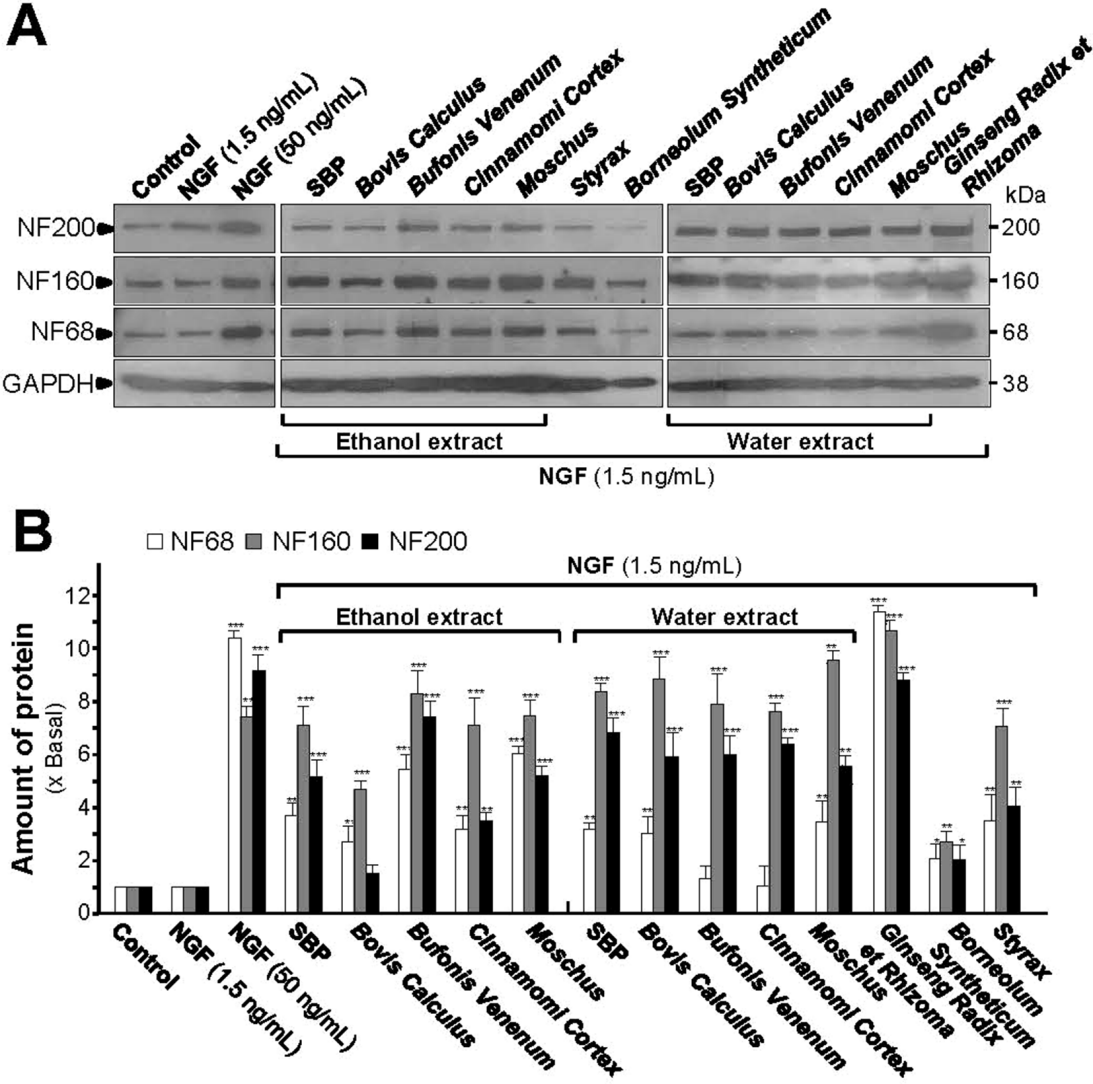
SBP extracts potentiate the NGF-induced expression of neurofilaments. **(A)**: Cultured PC12 cells were co-treated with NGF (1.5 ng/ml) together with SBP_water_ and SBP_EtOH_, or individual herbal extracts for 48 h. NGF at 50 ng/ml served as a positive control. SBP_EtOH_, ethanol extracts of various herbal materials and *Styrax* solution were used at 100 μg/ml; and SBP_water_, water extracts of various herbal materials, solutions of *Ginseng Radix et Rhizoma* extract and *Borneolum Syntheticum* were used at 500 μg/ml. The cell lysates were collected to determine the expression of NF68 (∼68 kDa), NF160 (∼160 kDa), and NF200 (∼200 kDa). GAPDH (∼38 kDa) served as a loading control. **(B)**: Quantification from the blots by a densitometer was shown. Values were expressed as the fold of change (x Basal) against the control (no treatment; set as 1), mean ± SEM, *n* = 4, * *p* < 0.05; ** *p* < 0.01; *** *p* < 0.001.

In low-dose NGF, both SBP_water_ and SBP_EtOH_ could potentiate the expression of NF68, NF160, and NF200 proteins (**Figures 4A, B**). Co-treatment of SBP_EtOH_ with low-dose NGF could induce the expressions of NF68, NF160, and NF200 by ∼4, ∼7, and ∼5 folds, respectively (**Figures 4A, B**). Co-treatment of SBP_water_ with 1.5 ng/ml NGF induced the expressions of NF68, NF160, and NF200 by ∼3, ∼8, and ∼7 folds, respectively (**Figures 4A, B**). In addition, the extracts of *Moschus*, *Ginseng Radix et Rhizoma*, *Cinnamomi Cortex*, *Bovis Calculus Artifactus*, *Styrax*, *Bufonis Venenum*, and *Borneolum Syntheticum* could differentially synergize with low-dose NGF to induce the expressions of neurofilaments. General speaking, the ethanol extracts showed better effect on protein expression of NF68 than the water extracts; while the water extracts showed stronger effect on protein expression of NF200. Among individual herbal materials, *Ginseng Radix et Rhizoma* extract showed the strongest capability to induce protein expressions of neurofilaments (**Figures 4A, B**).

### The SBP-Induced Neuronal Differentiation Is *via* PKA Signaling

**Figure 5:**
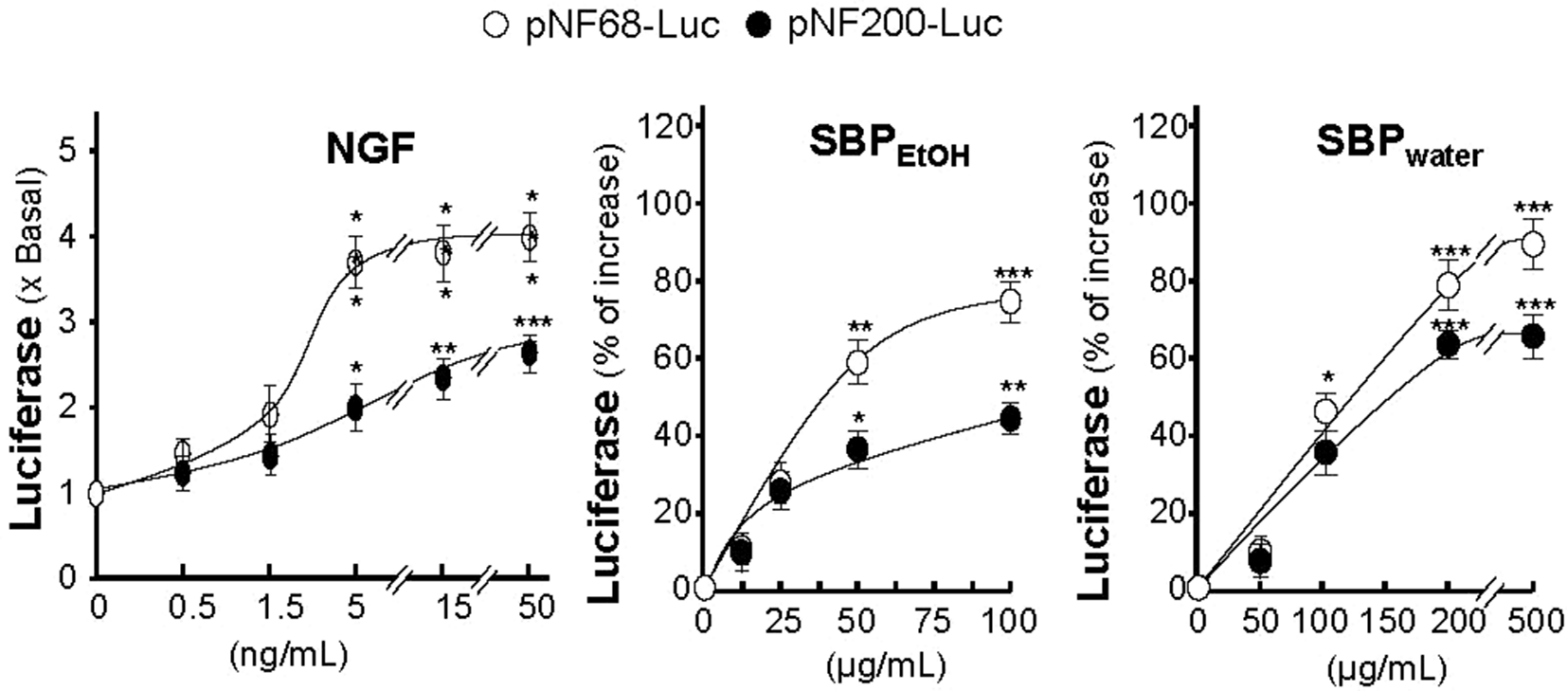
SBP extracts induce transcriptional activation of neurofilament promoters. Cultured PC12 cells were transfected with pNF68-Luc or pNF200-Luc and treated with different doses of NGF, SBP_water_, SBP_EtOH_, as indicated, for 48 h. The cell lysates were collected to determine the luciferase activity. Values are in mean ± SEM, *n* = 4, each with triplicate samples, * *p* < 0.05; ** *p* < 0.01; *** *p* < 0.001 as compared to the control group (no treatment).

The promoter–reporter constructs of neurofilaments, tagged with luciferase reporter gene (e.g., pNF68-Luc and pNF-200-Luc), were employed here, which were transfected into cultured PC12 cells. NGF, serving as a positive control, induced pNF68-Luc and pNF-200-Luc activities in dose-dependent manners (**Figure 5**). In parallel, the treatments of pNF68-Luc and pNF200-Luc transfected cultures with SBP_water_ or SBP_EtOH_ induced both NF68 and NF200 promoter activities in dose-dependent manners (**Figure 5**). The activation of neurofilament expression, triggered by SBP extracts, was more robust in NF68 than that of NF200.

**Figure 6:**
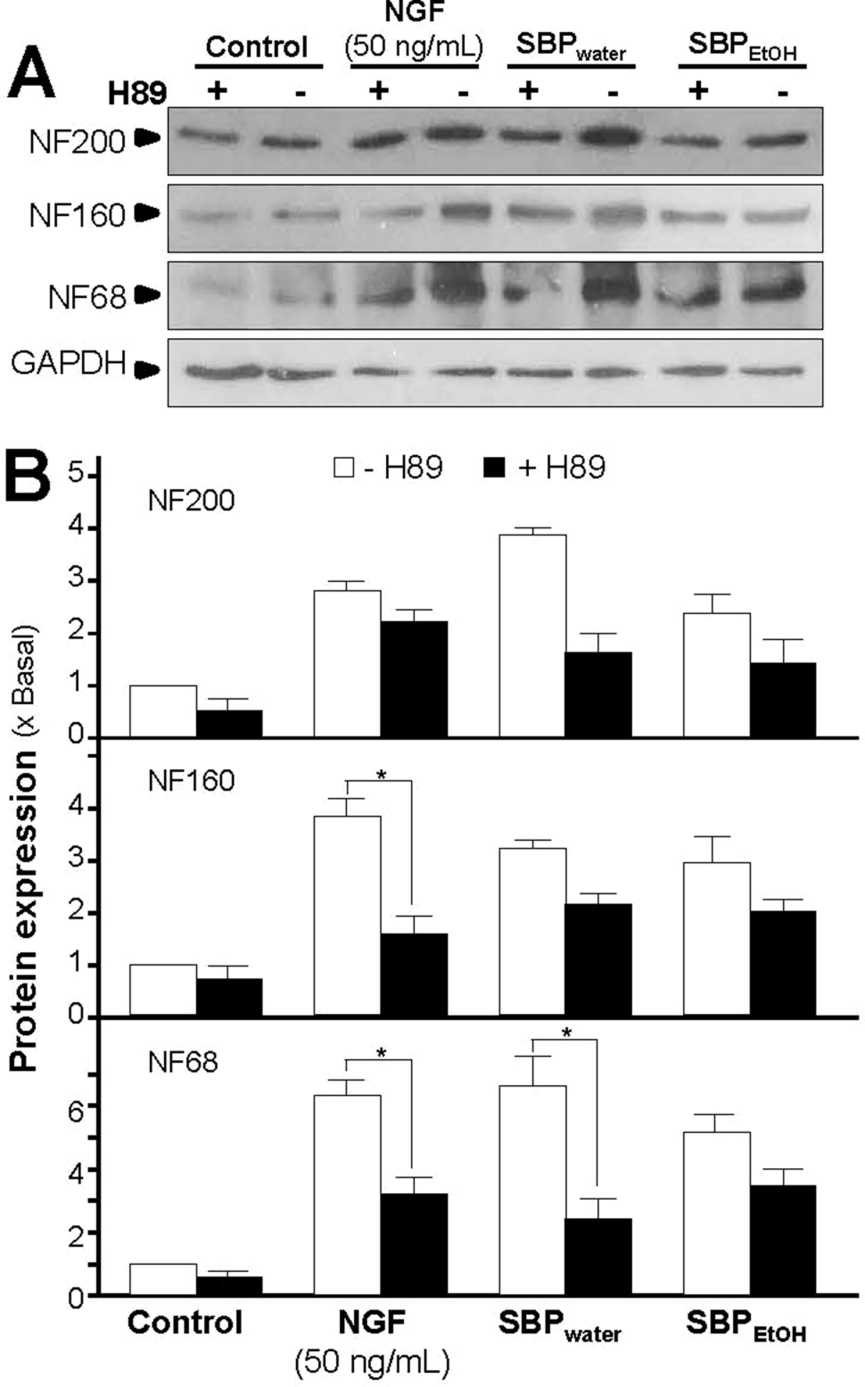
Inhibition of PKA partly suppresses SBP-induced expressions of neurofilaments. **(A)**: Cultured PC12 cells were pre-treated with or without PKA inhibitor, H89 (5 μM), for 3 h, and treated with SBP_water_ (500 μg/ml), or SBP_EtOH_ (100 μg/ml), or NGF at 50 ng/ml (positive control), for 48 h. The cell lysates were collected to determine the expressions of NF68 (∼68 kDa), NF160 (∼160 kDa), and NF200 (∼200 kDa). GAPDH (∼38 kDa) served as a loading control. **(B)**: Quantification from the blots by a densitometer was shown. Values were expressed as the fold of change (x Basal) against the control (no treatment; set as 1), mean ± SEM, *n* = 4, * *p* < 0.05.

Activation of cAMP signaling pathway is essential for neuronal differentiation of PC12 cells (Sánchez et al., 2004). An increase in intracellular level of cAMP was reported to induce neuronal differentiation, as well as cooperate with NGF in inducing PC12 cell neurite outgrowth (Richter-Landsberg and Jastorff, 1986). In cAMP signaling pathway, PKA is contributing to transcriptional control of neurofilament genes and neuronal differentiation (Sánchez et al., 2004). To investigate the potency of SBP extracts in stimulating neurite outgrowth and neurofilament expression *via* cAMP signaling pathway, PC12 cells were challenged with H89, a marketed selective PKA inhibitor, before the treatment of SBP_water_ or SBP_EtOH_. The application of H89 reduced the SBP-induced protein expressions of neurofilaments proteins, NF68, NF160, and NF200 (**Figure 6**). The reduction was robust and significant in the scenario of NF68 after treatment of SBP_water_. As a control, H89 blocked partially the NGF-induced neurofilament expression.

**Figure 7:**
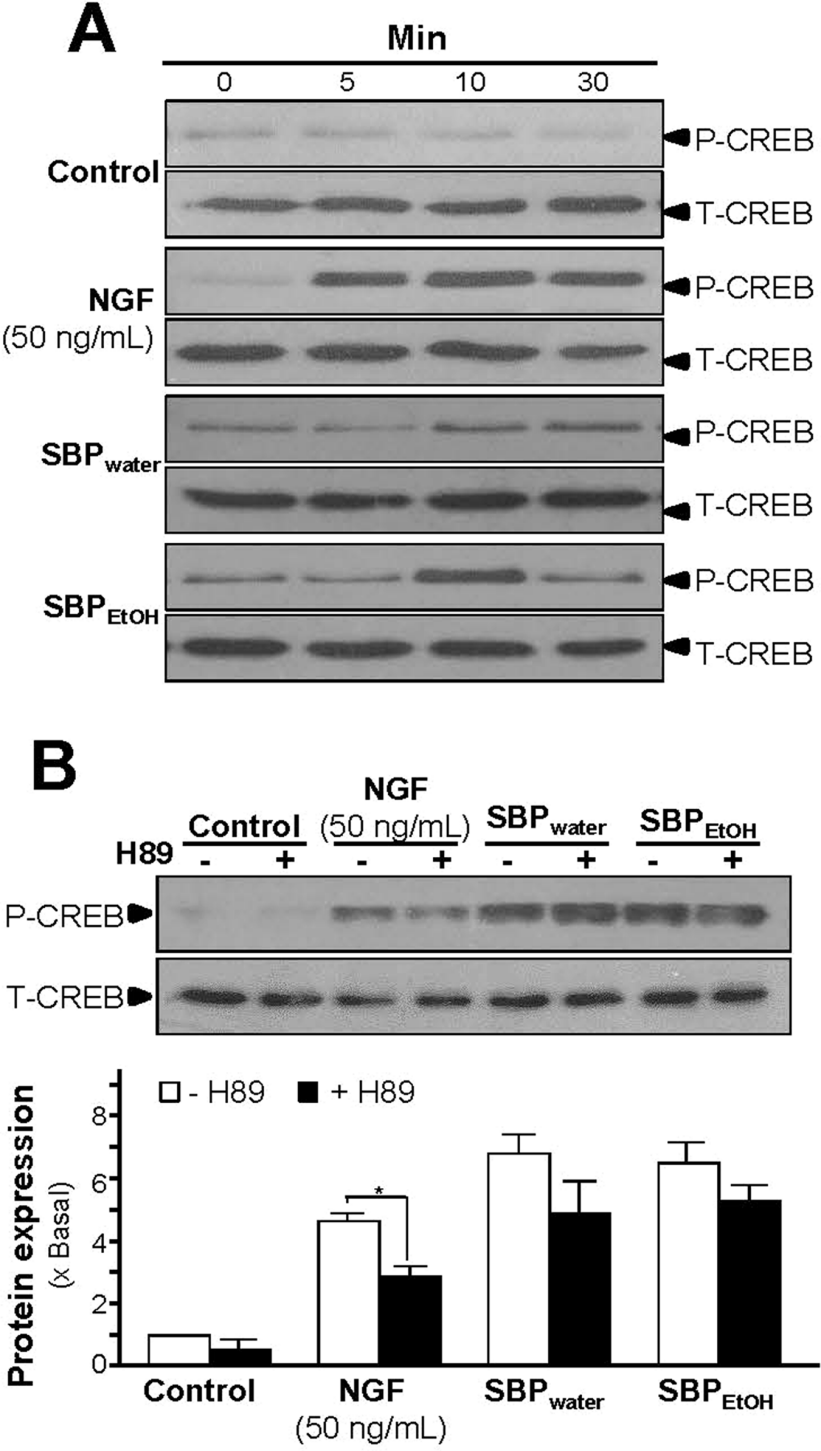
SBP extracts induce phosphorylation of CREB in PC12 cells. **(A)**: Serum starved PC12 cells were treated with SBP_water_ (500 μg/ml), or SBP_EtOH_ (100 μg/ml), or NGF at 50 ng/ml (positive control) for different time as indicated. The cell lysates were collected to determine the expressions of total CREB (∼42 kDa) and phosphorylated CREB (∼42 kDa), recognized by specific antibodies. **(B)**: Serum starved PC12 cells were pre-treated with or without PKA inhibitor, H89 (5 μM), for 3 h prior to the treatment with NGF, SBP_water_, or SBP_EtOH_ as in (A), for 10 min. Total CREB and phosphorylated CREB were identified (upper panel). Quantification from the blots by a densitometer was shown (lower panel). Values were expressed as the fold of change (x Basal) against the control (no treatment; set as 1), mean ± SEM, *n* = 4, * *p* < 0.05.

**Figure 8:**
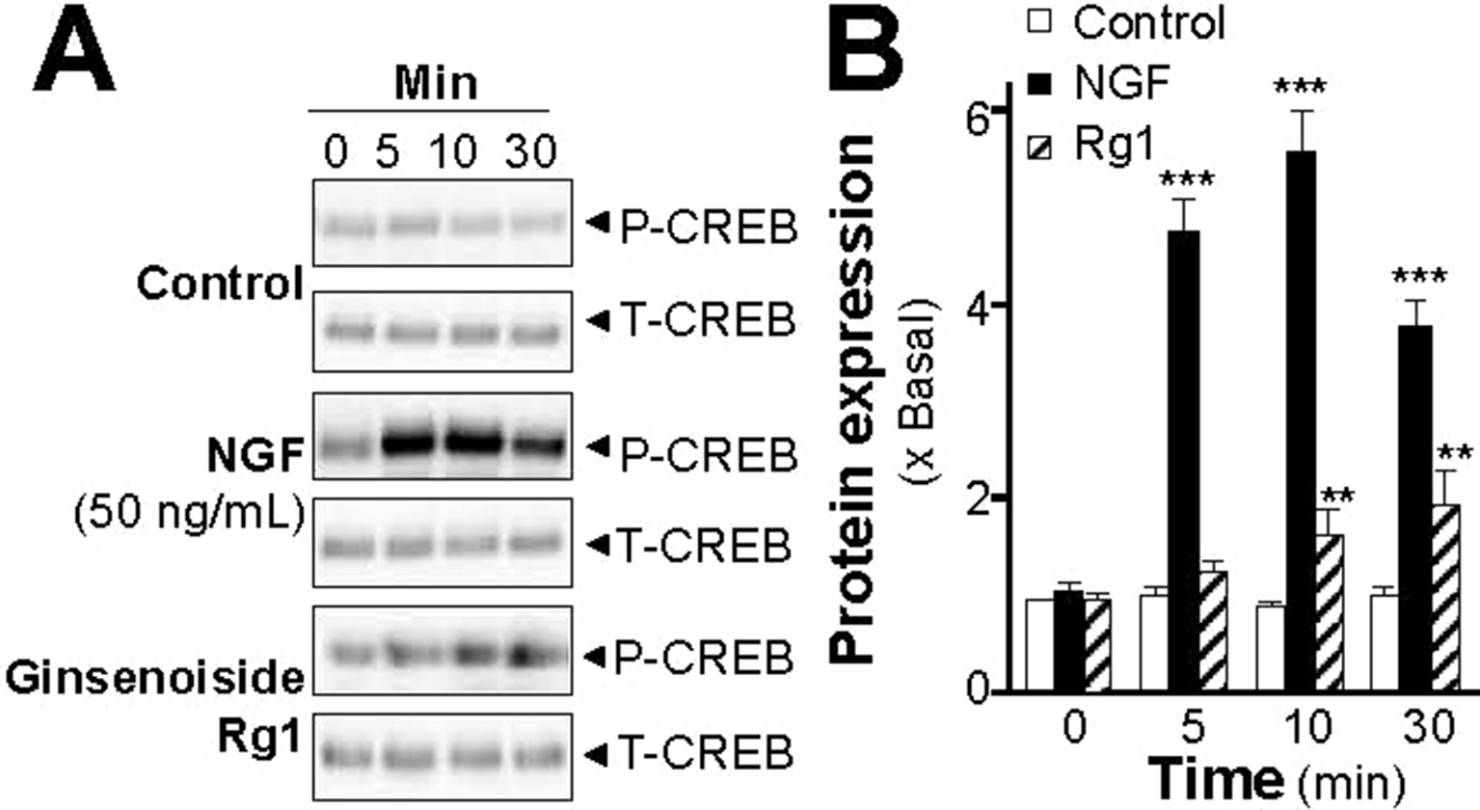
Ginsenoside Rg_1_ induces phosphorylation of CREB in PC12 cells. **(A)**: Serum starved PC12 cells were treated with ginsenoside Rg_1_ (10 μM), or NGF at 50 ng/ml (positive control), for different time as indicated. The cell lysates were collected to determine the expressions of total CREB (∼42 kDa) and phosphorylated CREB (∼42 kDa), recognized by specific antibodies. **(B)**: Quantification from the blots by a densitometer was shown. Values were expressed as the fold of change (x Basal) against the control (no treatment; set as 1), mean ± SEM, *n* = 4, ** *p* < 0.01; *** *p* < 0.001.

Increase of intracellular level of cAMP by activation of adenylate cyclase leads to activation of PKA that translocates into nucleus and phosphorylates transcriptional factor CREB. The phosphorylated CREB recruiting the coactivator allows its binding onto the promoter, and further stimulates the transcriptions of a series of target genes related to neuronal differentiation (Richter-Landsberg and Jastorff, 1986; Impey et al., 2004). Therefore, the phosphorylation of CREB was determined. NGF at 50 ng/ml robustly induced the phosphorylation of CREB in a time-dependent manner, serving as a positive control here (**Figure 7A**). The CREB phosphorylation, induced by the extracts of SBP, could be similar to that of NGF. The maximal induction of CREB phosphorylation at 10 min was at 6- to 7-folds in the treatments of SBP_water_ or SBP_EtOH_: this induction was reduced partly by application of a PKA inhibitor H89 (**Figures 7A, B**). The extract of *Ginseng Radix et Rizhoma* is one of the major components in the formulation of SBP. The extract of *Ginseng Radix et Rizhoma* could greatly induce neurite outgrowth and expressions of neurofilaments in cultured PC12 (**Figures 1**–**4**). Ginsenoside Rg_1_, a steroidal saponin of high abundance in ginseng, is known to possess the neuroprotective effects. Here, the role of ginsenoside Rg_1_ on the phosphorylation of CREB was further evaluated. As expected, the maximal induction of CREB phosphorylation at 30 min was at ∼2 folds under the treatments of 10 μM ginsenoside Rg1 (**Figures 8A, B**).

**Figure 9:**
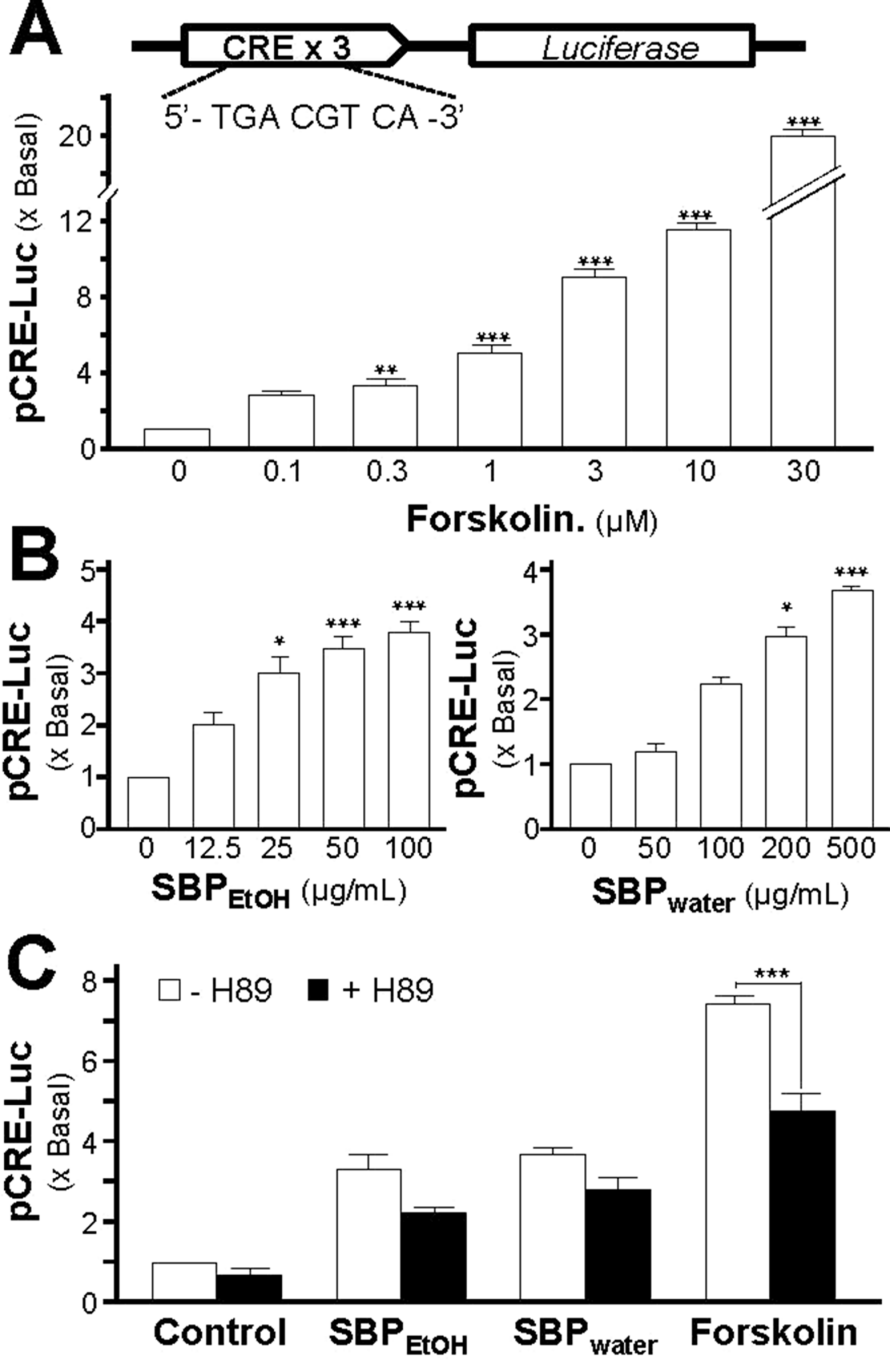
SBP induces the cAMP-mediated transcriptional activity in PC12 cells. **(A)**: Cultured PC12 cells, transfected with pCRE-Luc, were treated with of forskolin, as indicated for 48 h. The cell lysates were collected to determine the luciferase activity. **(B)**: The pCRE-Luc transfected PC12 cells were treated with SBP_water_ or SBP_EtOH_, as indicated for 48 h. **(C)**: The pCRE-Luc transfected PC12 cells were pre-treated with PKA inhibitor, H89 (5 μM), for 3 h, and then treated with or SBP_EtOH_ (100 mg/ml), or SBP_water_ (500 μg/ml), or forskolin (3 μM) for 48 h. The cell lysates subjected to luciferase assay. Values are expressed as the fold of increase to basal reading, and are in mean ± SEM, *n* = 4, each with triplicate samples. * *p* < 0.05; ** *p* < 0.01; *** *p* < 0.001.

The adenylate cyclase activator, forskolin, raises intracellular cAMP level in PC 12 culture; this induction of forskolin on neuronal differentiation could be inhibited by H89 (Lam et al., 2016b). To investigate the induction effect of SBP_water_ or SBP_EtOH_ on transcriptional activation of CRE, a luciferase-reporter construct (pCRE-Luc), containing three copies of CRE derived from the promoter, and tagged with luciferase reporter gene, was transfected into PC12 cells. Forskolin, a positive control, induced pCRE-Luc activity in a dose-dependent manner: the maximal induction was at over 20 folds at 30 µM (**Figure 9A**). SBP_water_ or SBP_EtOH_ induced the pCRE-Luc activity in dose-dependent manners (**Figure 9B**). The maximal induction could reach about 4 folds. To further confirm the signaling triggered by SBP_water_ or SBP_EtOH_, the pCRE-Luc transfected PC12 cells were pre-treated with H89: the SBP-induced transcriptional activity of pCRE-Luc was fully blocked by application of H89 (**Figure 9C**). This result indicated the function of the extracts of SBP in neuronal differentiation possibly related to cAMP-dependent signaling.

## Discussion

SBP is a well-known composite formula of TCM in treating cardiovascular diseases (Yan et al., 2006), which has efficacy in the treatment of angina pectoris and chest pain, caused by coronary heart diseases (Hong et al., 2006; Yan et al., 2006; He et al., 2009; Tian et al., 2011). Several lines of evidence suggested possible new function of SBP in brain disease i.e., treating cerebral ischemia reperfusion injury (Chen et al., 2008). In chronic stress mice, the intake of SBP could reverse the stress-suppressed levels of neurotransmitters’ metabolites and neurotrophic factors in the brain (Zhou et al., 2019). These therapeutic effects of SBP in brain could be related to the properties of SBP in cytoprotection and immunomodulation (Zhou et al., 2016; Zhang et al., 2017). Here, we are proposing a new potential application of SBP in treating neurodegenerative diseases. This notion is being supported by the evidence in SBP-treated cultured PC12 cells: (i) SBP together with low-dose NGF inducing neurite outgrowth; (ii) SBP alone or together with low-dose NGF inducing expression of neurofilament; and (iii) SBP inducing phosphorylation of CREB. To pharmacologically test the role of SBP on neuronal differentiation in PC12 cells, both water (SBP_water_) and 95% ethanol (SBP_EtOH_) extracts of SBP were used in current study. Water extraction is the most common method in the preparation of herbal decoctions; while 95% ethanol is used to extract the polar and semi-polar constituents of SBP. The solvent-caused difference in bioactivities of SBP and each material may contribute to future modification of SBP formula for preventing neurodegenerative diseases.

Neurogenesis is vital for spatial learning in rodents and primates (Seib and Martin-Villalba, 2015). A stimulation of neurogenesis is to speed up the recovery processes during neurodegenerative diseases (Costa et al., 2015). In the brain, neurotrophic factors promote neurogenesis and maturation of progenitor cells (Urbán and Guillemot, 2014). Among these neurotrophic factors, NGF is one of the key modulators for neurogenesis, neurite outgrowth, nerve plasticity, and axonal outgrowth during neuronal development, and indeed many neurodegenerative diseases are closely associated with insufficiency of NGF in the brain, e.g., depression and Alzheimer’s disease (Xu et al., 2013; Xu et al., 2016). In neuron, NGF achieves its function by binding and activating TrkA receptor, which results in neuronal differentiation and promoting cell survival (Uren and Turnley, 2014). NGF could induce neurite outgrowth of PC12 cell and N2a cell in a dose-dependent manner. Low-dose of NGF at 1.5 ng/ml failed to induce the neurite extension. The notion is supported by the effect of low-dose of NGF on neurite outgrowth in both PC12 cell (Yan et al., 2017) and N2a cell (Phan et al., 2013). Therefore 1.5 ng/ml NGF was chosen for the co-treatment analyses. The percentage of neurite bearing cells increased significantly in PC12 cell and N2a cell, treated with 50 ng/ml NGF, when compared to negative control (Phan et al., 2013; Lam et al., 2017; Yan et al., 2017). Based on these findings, the optimized concentration of NGF (50 ng/ml) was selected as a positive control.

Neurite outgrowth is a key process during neuronal development, migration, and differentiation (Khodosevich and Monyer, 2010; Park et al., 2017). Neurofilaments are the major cytoskeletal elements in neurite, which accumulate in many neurological diseases, e.g., Charcot-Marie-Tooth disease, Parkinson’s disease, and amyotrophic lateral sclerosis (Al-Chalabi and Miller, 2003). Here, the extracts of SBP could synergize with a low level of NGF in stimulating neurite outgrowth, as well as a direct induction of neurite outgrowth. In addition, the induction effect of SBP extracts on expressions of neurofilaments (NF68, NF160, and NF200) was fully supported with or without NGF. In parallel, the transcriptional activities of NF68/NF200 in cultured PC12 cells could be triggered SBP. In the SBP-induced PC12 cell differentiation assays, SBP_EtOH_ in general showed better effects, as compared to that of SBP_water_, suggesting the effective chemicals should be more soluble in ethanol. This notion is fully supported by HPLC fingerprints of SBP extracts, i.e. more peaks being identified in SBP_EtOH_ than that of SBP_water_. Thus, the intake of SBP may relieve neurodegenerative diseases in suffering from a deficiency of NGF in the brain; however, this notion has to be verified by animal study.

Among the medicinal materials in SBP, *Moschus* is a rare medicinal herb commonly used in treating damaged brain. *Borneolum Synthcticum* is regularly used for stroke treatment due to its refreshing and resuscitating nature. The combination of these two constituents is considered as a drug-pair in clinical application (Xia et al., 2014). This drug-pair prevented neurons from apoptosis and relieved the brain damage. *Bovis Calculus Artifactus* has been used for more than 2,000 years in TCM practice, and one of the major components is the oxygenated derivative of cholesterol-oxysterol, a chemical being shown to possess neuroprotective effects (Wang et al., 2015). *Cinnamomi Cortex* showed brain protective function by increasing the activity of superoxide dismutase and decreasing the oxidative stress (Lv, 2015). Cinofufagin and resibufogenin, main constituents in *Bufonis Venenum*, have been shown to be involved in excitatory action in central nervous system (Bi et al., 2016). *Styrax* is applied in treatment of acute stroke because of it resuscitation-inducing nature (Zhang et al., 2019). Our studies here are in good agreement with previous results that the medicinal constituents in SBP should have capability in inducing neurite outgrowth and neuroprotection (Xia et al., 2014; Zhang et al., 2019). The bioactive components of SBP distributing into the brain through the BBB provides the basis for the effect of SBP in treating brain disorders. Muscone, the major bioactive component of *Moschus*, could pass through rat BBB and soon reached the highest peak in the brain. In addition, it is reported that muscone in the brain remains at higher concentration, and metabolizes much slower when compared to other organs (Chen et al., 2004). Ginsenosides could cross the BBB, and ginsenoside Rg_1_ is the main component that entered the brain after oral administration of ginseng total saponins (Zhao et al., 2018). *Cinnamomi Cortex* could be metabolized to sodium benzoate in the liver, and the metabolite could pass through the BBB (Jana et al., 2013). Recent pharmacological studies have shown that aromatic refreshing TCMs, such as *Borneolum Syntheticum*, *Moschus*, and *Styrax*, could induce resuscitation and modify the permeability of the BBB, promoting the entry of other drugs into the brain with brain protective effects (Lv et al., 2018). The component of *Bufonis Venenum* (Kou et al., 2014), as well as bile acid and taurocholic acid of *Bovis Calculus Artifactus* (Kitazawa et al., 1998; Quinn et al., 2014), were reported to possess the ability to pass through the BBB. The findings pave a direction of SBP having clinical application in neurodegenerative disorders.

The involvement of PKA-CREB signaling was shown in SBP-induced neuronal differentiation, at least in cultured PC12 cells. Application of SBP induced the transcriptional activities of pNF68/200-Luc and pCRE-Luc, expression of neurofilaments, and phosphorylation of CREB. In parallel, the synergistic effect of SBP could boost the NGF’s function in neuronal differentiation. The inhibition of PKA signaling by H89 partially suppressed the SBP-induced neuronal differentiation, implying that: (i) the ingredients of SBP extracts could penetrate cell membrane and directly activate intracellular signaling molecules; (ii) other players, such as EPAC (exchange proteins directly activated by cAMP), might possibly be involved; and (iii) SBP extracts might indirectly potentiate NGF-induced signaling by increasing the NGF binding affinity to Trk A receptor. In addition, ginsenoside Rg1, as one of the major bioactive components in SBP, contributed to the activation of PKA-CREB signaling and neuronal differentiation in PC12 cells. SBP is a herbal mixture containing numerous varieties of chemicals, and each of them functionally could be rather different. Besides, synergy among these phytochemicals has to be considered.

## Conclusion

Neurodegenerative diseases could be a cause triggered by deficiency of NGF in the brain. SBP, a commonly used TCM in the market today, is found to possess trophic activity in modulating neuronal differentiation in PC12 cells. The induction effect of SBP extracts in expression of neurofilaments is mediated by a cAMP-PKA signaling pathway. Therefore, the SBP formulation might be considered as a new direction in developing promising drug, or health food supplement, in treating and/or preventing neurodegenerative diseases.

## Data Availability Statement

All datasets generated for this study are included in the manuscript and the **Supplementary Files**.

## Author Contributions

MX, Z-YZ, Y-JX, EL and W-HH performed the experiments. SC and RD prepared and analysed the water and ethanol extracts of SBP. X-HS and C-SZ performed the HPLC fingerprint analysis and supplied the materials. MX and KT designed and analyzed the data and revised the manuscript. TD and KT organized and supervised the study. All authors read and approved the final manuscript.

## Funding

This study was supported by Chinese Association of Integrated Traditional and Western Medicine-SHPL Research Fund No. 2018001, Hong Kong RGC Theme-based Research Scheme (T13-607/12R), Innovation Technology Fund (UIM/340, UIM/385, ITS/500/18FP), HMRF18SC06, Shenzhen Science and Technology Committee Research Grant (CKFW2, 016, 082, 916, 015, 476; JCYJ20,180,306,174,903,174; JCYJ20, 170, 413, 173, 747, 440; JCYJ20, 160, 229, 205, 726, 699; JCYJ20, 160, 229, 205, 812, 004; JCYJ20, 160, 229, 210, 027, 564; ZDSYS201, 707, 281, 432, 317, and 20, 170, 326).

## Conflict of Interest

C-SZ and X-HS were employed by Shanghai Hutchison Pharmaceuticals Ltd. and the Shanghai Engineering Research Center for Innovation of Solid Preparation of TCM.

The remaining authors declare that the research was conducted in the absence of any commercial or financial relationships that could be construed as a potential conflict of interest.

## Abbreviations

SBP, Shexiang Baoxin Pill; BBB, Blood-brain barrier; TCM, Traditional Chinese Medicine; NGF, Nerve growth factor; AD, Alzheimer’s disease; NTFs, Neurotrophic factors; PKA, Protein kinase A; CREB, cAMP responsive element binding protein; DMEM, Dulbecco’s modified Eagle’s medium; HRP, Horseradish peroxidase; CMV, Cytomegalovirus; DTT, Dithiothreitol; MDA, Malondialdehyde.

## Supplementary Material

Figure S1 HPLC fingerprint of SBP and other herbal extracts. Chemical compositions of water and 95% ethanol extracts of SBP, *Bufonis Venenum*, *Bovis Calculus*, *Moschus* and *Cinnamomi Cortex* were analyzed by HPLC. The chromatographic method was described in materials and methods section. The identities of peaks are: (1) benzoic acid from *Styrax*; (2) cinnamic acid from *Styrax* and *Cinnamomi Cortex*; (3) cinnamaldehyde from *Styrax* and *Cinnamomi Cortex*; (4) cinobufagin from *Bufonis Venenum*; (5) recibufogenin from *Bufonis Venenum*; (6) ethyl cinnamate from *Styrax*; (7) benzyl benzoate from *Styrax*; (8) benzyl cinnamate from *Styrax*; (9) phenethyl cinnamate from *Styrax*; (10) cinnamyl cinnamate from *Styrax*; (11) unknown from *Styrax*; and (12) unknown from *Styrax*. Typical profiles were shown, where *n* = 4.

Figure S2 HPLC fingerprint of *Ginseng Radix et Rhizoma*, *Borneolum Syntheticum* and *Styrax*. *Ginseng Radix et Rhizoma*, *Borneolum Syntheticum* and *Styrax* were supplied with extract or crystalized powder, as stated in Chinese Pharmacopeia 2015. *Ginseng Radix et Rhizoma* was supplied as dried 75% ethanol extract of *P. ginseng* root and rhizome, having over 0.27% ginsenoside Rg_1_ and ginsenoside Re and ginsenoside Rb_1_ not less than 0.18% by weight, *Borneolum Syntheticum* was supplied as synthetic crystal containing mainly borneol not less than 55% by weight. *Styrax* was supplied as acaroid resin obtained from the trunk of *L. orientalis* having over 5% cinnamic acid by weight. These dried powders were dissolved in DMSO for HPLC analyses. The chromatographic method was described in materials and methods section, as in Supplementary figure 1. The identities of peaks are: (1) benzoic acid from *Styrax*; (2) cinnamic acid from *Styrax* and *Cinnamomi Cortex*; (3) cinnamaldehyde from *Styrax* and *Cinnamomi Cortex*; (4) cinobufagin from *Bufonis Venenum*; (6) ethyl cinnamate from *Styrax*; (8) benzyl cinnamate from *Styrax*; (9) phenethyl cinnamate from *Styrax*; (10) cinnamyl cinnamate from *Styrax*; (11) unknown from *Styrax*; and (12) unknown from *Styrax*. Typical profiles were shown, where *n* = 4.
