# Supplementary figures and images for: Shexiang Baoxin Pill, a Formulated Chinese Herbal Mixture, Induces Neuronal Differentiation of PC12 Cells: A Signaling Triggered by Activation of Protein Kinase A
Source: Front Pharmacol. 2019 Oct 9;10:1130. doi: 10.3389/fphar.2019.01130 (PMC6794430; doi:10.3389/fphar.2019.01130)

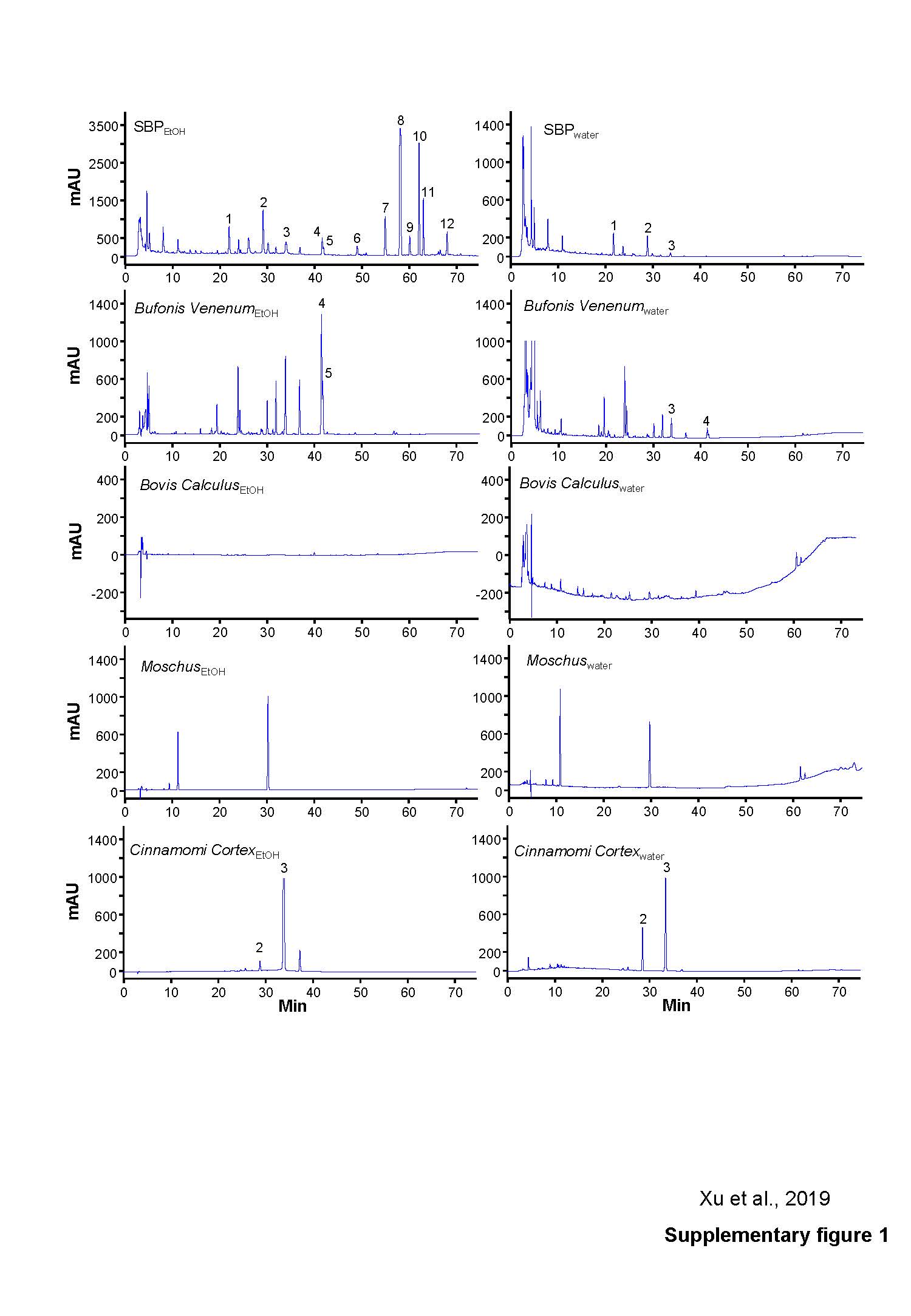

Supplement: Figure S1 — HPLC fingerprint of SBP and other herbal extracts. Chemical compositions of water and 95% ethanol extracts of SBP, Bufonis Venenum, Bovis Calculus, Moschus and Cinnamomi Cortex were analyzed by HPLC. The chromatographic method was described in materials and methods section. The identities of peaks are: (1) benzoic acid from Styrax; (2) cinnamic acid from Styrax and Cinnamomi Cortex; (3) cinnamaldehyde from Styrax and Cinnamomi Cortex; (4) cinobufagin from Bufonis Venenum; (5) recibufogenin from Bufonis Venenum; (6) ethyl cinnamate from Styrax; (7) benzyl benzoate from Styrax; (8) benzyl cinnamate from Styrax; (9) phenethyl cinnamate from Styrax; (10) cinnamyl cinnamate from Styrax; (11) unknown from Styrax; and (12) unknown from Styrax. Typical profiles were shown, where n = 4. [file Image_1.jpeg]

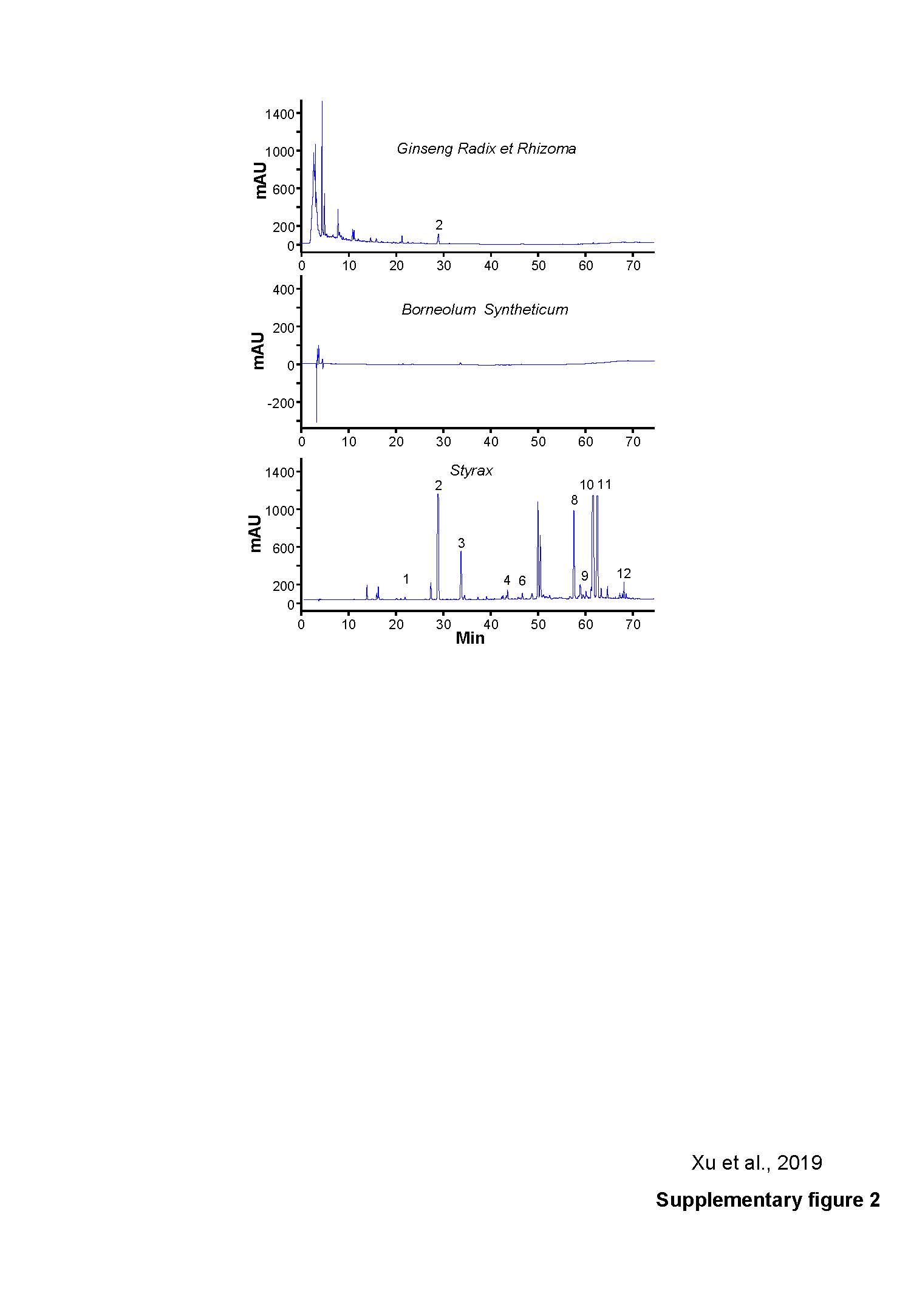

Supplement: Figure S2 — HPLC fingerprint of Ginseng Radix et Rhizoma, Borneolum Syntheticum and Styrax. Ginseng Radix et Rhizoma, Borneolum Syntheticum and Styrax were supplied with extract or crystalized powder, as stated in Chinese Pharmacopeia 2015. Ginseng Radix et Rhizoma was supplied as dried 75% ethanol extract of P. ginseng root and rhizome, having over 0.27% ginsenoside Rg1 and ginsenoside Re and ginsenoside Rb1 not less than 0.18% by weight, Borneolum Syntheticum was supplied as synthetic crystal containing mainly borneol not less than 55% by weight. Styrax was supplied as acaroid resin obtained from the trunk of L. orientalis having over 5% cinnamic acid by weight. These dried powders were dissolved in DMSO for HPLC analyses. The chromatographic method was described in materials and methods section, as in Supplementary figure 1. The identities of peaks are: (1) benzoic acid from Styrax; (2) cinnamic acid from Styrax and Cinnamomi Cortex; (3) cinnamaldehyde from Styrax and Cinnamomi Cortex; (4) cinobufagin from Bufonis Venenum; (6) ethyl cinnamate from Styrax; (8) benzyl cinnamate from Styrax; (9) phenethyl cinnamate from Styrax; (10) cinnamyl cinnamate from Styrax; (11) unknown from Styrax; and (12) unknown from Styrax. Typical profiles were shown, where n = 4. [file Image_2.jpeg]
